# Aerosol inhalation of human IFN-α1b exhibits anti-RSV activity in mice and favorable pharmacokinetics/safety in cynomolgus monkeys

**DOI:** 10.3389/fmicb.2026.1767720

**Published:** 2026-04-30

**Authors:** Mingming Yang, Liping Guo, Yanran Wang, Jiangtao Bai, Xiaowen Liang, Jiahua Kuang, Hongjie Ma, Yang Yang

**Affiliations:** 1 Shenzhen Kexing Pharmaceutical Co., Ltd, Shenzhen, China; 2 Department of Microbiology, School of Basic Medicine, Guangxi Medical University, Nanning, China; 3 Capital Center for Children’s Health, Capital Medical University, Beijing, China; 4 Shenzhen Key Laboratory of Pathogen and Immunity, Shenzhen Clinical Research Center for Infectious Disease, State Key Discipline of Infectious Disease, Shenzhen Third People's Hospital, Second Hospital Affiliated to Southern University of Science and Technology, Shenzhen, China

**Keywords:** aerosol inhalation, cynomolgus monkey, IFN-α1b, pharmacokinetics, respiratory syncytial virus

## Abstract

**Introduction:**

Respiratory syncytial virus (RSV) is a leading cause of lower respiratory tract infections, while effective therapeutic options remain limited. This study investigated the antiviral efficacy, pharmacokinetics, and safety of aerosolized human interferon-α1b (IFN-α1b) as a potential treatment for RSV infection.

**Methods:**

The antiviral activity of IFN-α1b against RSV was evaluated *in vitro* and in an RSV-infected mouse model. Pharmacokinetics, tissue distribution, and repeat-dose inhalation toxicity were assessed in cynomolgus monkeys.

**Results:**

IFN-α1b inhibited RSV *in vitro* (EC₅₀ of 4.64 ng/mL). In mice, aerosolized IFN-α1b reduced lung viral titers in a dose-dependent manner. Notably, the reduction in viral load across all IFN-α1b dose groups was greater than that observed in the ribavirin-treated group under the conditions tested (*p* < 0.001). In monkeys, inhalation resulted in high respiratory drug exposure with an absolute systemic bioavailability of 3.33%. Repeated dosing (18 μg/kg/day) was well tolerated, with only minimal to mild (Grade 1 to 2) focal lung inflammation that was fully reversible.

**Conclusion:**

Aerosolized IFN-α1b demonstrates antiviral activity and favorable lung-targeted pharmacokinetics in preclinical models, supporting further investigation in clinical settings. It was associated with greater reduction in lung viral titers compared with ribavirin in this model while maintaining a favorable safety profile.

## Background

Respiratory Syncytial Virus (RSV), a negative-sense single-stranded RNA virus (family Pneumoviridae), remains the major cause of acute respiratory tract infections in infants and young children worldwide (Li et al., 2022). Clinical symptoms range from mild upper respiratory infections to severe lower respiratory infections, such as bronchiolitis and pneumonia (Pratt et al., 2022). Most children are infected with RSV in the first 2 years, with reinfections occurring throughout life (Foley et al., 2023). RSV caused 3.33 million acute lower respiratory tract infections and 26,300 deaths in children under 5 years globally (Li et al., 2022). It accounts for 2% of all under-5 deaths and ~14,000 annual deaths in adults aged ≥65 (Yang et al., 2025). Notably, in addition to children, RSV has also been increasingly recognized as a cause of illness in adults, particularly among the elderly (≥65 years of age) and high-risk populations like those with chronic obstructive pulmonary disease (COPD) and congestive heart failure (Shook and Lin, 2017). As a respiratory virus, RSV is primarily transmitted between individuals via saliva or mucus droplets. The viral load is correlated with disease severity, similar to many other common respiratory viruses (Shang et al., 2021; Utokaparch et al., 2011; DeVincenzo et al., 2010). Despite typically causing mild-to-moderate illness, RSV imposes substantial healthcare burdens, particularly in developing regions (Griffiths et al., 2017; Nair et al., 2010).

Current interventions include vaccines, monoclonal antibodies (mAbs), and antivirals (Simões et al., 2015; Bergeron and Tripp, 2020; Fearns and Deval, 2016; Mejias and Ramilo, 2015). RSV vaccines, Arexvy, ABRYSVO, and mRESVIA, were approved by the US FDA in May 2023, June 2023, and May 2024, respectively. These vaccines are limited to the prevention of lower respiratory diseases caused by RSV infection in adults over 60 years old (Yang et al., 2025; Fleming-Dutra et al., 2023; Sevendal et al., 2024). The monoclonal antibody for the prevention of RSV disease in infants, Beyfortus (nirsevimab), was approved by the FDA in June 2023 (Yang et al., 2025). In addition, Gao et al. reported the isolation of two neutralizing mAbs against RSV from convalescent children using the prefusion form of fusion (F) glycoprotein as bait. One mAb, RV11, exhibited good potency in neutralizing RSV strains from both A and B subtypes in a cell-based assay and protected mice from RSV infection *in vivo* (Dai et al., 2023). The FDA-approved drug for RSV treatment is ribavirin, a broad-spectrum antiviral agent. However, its application is constrained by complex administration routes, potential toxicity concerns, and insufficient evidence of efficacy. As such, its use is currently restricted to high-risk populations, including hematopoietic stem cell transplant (HSCT) recipients and lung transplant patients (Griffiths et al., 2017; Mejias and Ramilo, 2015; De Clercq and Li, 2016). The development of RSV-specific antivirals remains relatively limited, although EDP-938 and AK0529 have reported promising phase 2 efficacy and safety data (Sevendal et al., 2024). Given these limitations, there is a growing need for alternative RSV treatments that offer improved cost-effectiveness and therapeutic outcomes.

Interferons (IFNs) are critical components of the host’s antiviral defense mechanisms, activating key pathways that help control and eliminate viral infections. In the context of RSV infections, the role of Type I IFNs is particularly significant. Studies have shown that enhancing Type I IFN responses can reduce disease severity and improve outcomes in RSV infections. For instance, in neonatal mouse models, the administration of IFN-*α* prior to RSV infection has been demonstrated to reduce immunopathology and improve viral clearance (Hijano et al., 2019). Neonatal mice, for example, cannot produce IFN-α during RSV infection due to inadequate plasmacytoid dendritic cell (pDC) recruitment (Hijano et al., 2019). However, there are limitations in evaluating the effects of IFN-α because of its low species cross-reactivity. IFN-α1b is a broad-spectrum human interferon that effectively stimulates the immune system’s antiviral response. A clinical study evaluating IFN-α1b treatment for neonatal RSV pneumonia was conducted. The duration of symptoms was significantly shorter in the treatment group compared to the control group, indicating that IFN-α1b is both effective and safe for the treatment of neonatal RSV pneumonia (He et al., 2020). Moreover, another study focused on the impact of recombinant human IFN-α1b treatment on subsequent wheezing episodes in infants hospitalized with lower respiratory tract infections. The findings suggested that early use of IFN-α1b could reduce the incidence of wheezing episodes within the following year, indicating its protective role against recurrent respiratory issues (Yang et al., 2021). Nevertheless, subsequent information regarding the clinical application and progress of IFN-α1b in RSV treatment remains scarce.

In this study, a murine model of RSV infection was used to evaluate the *in vivo* antiviral efficacy of human IFN-α1b using a dose-escalation design with intratracheal aerosol administration. In parallel, pharmacokinetic properties were assessed in cynomolgus monkeys following both intravenous and inhalation administration. In addition, tissue distribution after single-dose inhalation and safety following repeated inhalation at multiple dose levels were evaluated in the non-human primate model to characterize lung-targeted delivery and tolerability.

## Materials and methods

### CCK-8 cell viability and anti-RSV activity assays

To evaluate cell viability and antiviral efficacy, HEp-2 cells were seeded into 96-well plates at 6,000 cells per well and incubated for 24 h. A unified 5-day window was employed for both cytotoxicity and antiviral assays, which were conducted in parallel to ensure synchronized drug exposure. For the cytotoxicity assay, cells were treated with serial dilutions of IFN-α1b (ranging from 1,333 to 0.017 ng/mL) or ALS-8112 (100 to 0.001 μM) and incubated for 5 days. In the simultaneous antiviral assay, HEp-2 cells were first infected with the RSV Long strain (MOI 0.01) for 2 h at 37 °C. Immediately following this adsorption period, the viral inoculum was removed and replaced with medium containing the identical serial dilutions of IFN-α1b or ALS-8112. The cells were then incubated for 5 days post-infection (dpi). At the conclusion of this 5-day period, cell viability and the inhibition of the viral cytopathic effect (CPE) were quantified using the CCK-8 assay. At the time of treatment (24 h post-seeding), cell confluency was approximately 30–40%, and untreated cells typically reached near-confluence within 72 h.

### Evaluation of the antiviral activity of IFN-α1b against RSV in mouse models

A total of 60 female BALB/c mice, aged 6–7 weeks and weighing 18–20 g, were randomly divided into 5 groups (12 mice per group) (Shanghai Jihui Experimental Animal Breeding Co., Ltd., China). Each mouse was intranasally inoculated with the RSVA strain Long (GenBank ID: KF713490.1) at a dose of 10^5^ plaque-forming units (PFU). From day 0 to day 4 post-inoculation, each group of mice received the corresponding test drugs via intratracheal aerosol administration once daily, with the first dose administered 1 h prior to virus inoculation. Mice were anesthetized, and a microsprayer device was inserted into the trachea to deliver aerosolized formulation directly into the lungs. For aerosol administration, IFN-α1b was nebulized from a prepared solution, and the indicated volume (50 μL) refers to the formulation volume rather than an intranasal instillation dose. The first group received 50 μL/animal of solvent as a control, the second group received 1.0 mg/animal ribavirin, and the third to fifth groups received human IFN-α1b dissolved in 50 μL of solvent at doses of 0.4, 2, and 10 μg/animal, respectively. On day 5, all animals were euthanized by CO2 inhalation, and lung tissues were collected and stored at −80 °C for subsequent plaque assays to determine viral titers. All mouse study protocols were conducted at WuXi AppTec and approved by its Institutional Animal Care and Use Committee (IACUC; Approval No.: ID01-QD029-2020v1.0). All procedures were performed in accordance with relevant institutional guidelines and regulations for animal welfare. Dose selection for the mouse efficacy study was based on a dose-escalation design to characterize the *in vivo* dose–response relationship of IFN-α1b against RSV. The selected doses (0.4, 2, and 10 μg/animal) were chosen to cover a range from the minimum effective dose to near-maximal antiviral activity, as determined by lung viral titer reduction.

### Plaque assay of RSV

The lung tissue homogenates obtained from mice were subjected to serial dilution in DMEM. Subsequently, Hep-2 cells cultured in 12-well plates were inoculated with 0.05 mL of the tissue homogenate supernatant at 37 °C for a duration of 2 h. During this period, gentle agitation was performed every 15 min to guarantee uniform exposure. After the incubation period, the inoculum was substituted with an overlay medium containing 1.2% agarose and 2% fetal bovine serum (FBS). The cells were then incubated for an additional 7 days under standard culture conditions. Subsequently, the overlays were meticulously removed, and plaques were made visible by staining with a 0.1% crystal violet solution. Viral titers were presented as PFU/g lung tissue.

### Pharmacokinetics of human IFN-α1b in cynomolgus monkeys

Thirty cynomolgus monkeys (15 males and 15 females, aged 2.8 to 3.5 years, weighing 2.20 to 3.34 kg) with similar body weights were selected. The animals were randomly divided into five groups based on sex, with three males and three females in each group. Animals in Group 1 were administered human IFN-α1b intravenously at a dose of 18 μg/kg. Serum samples were collected prior to administration and at the following time points post-administration: 2 min, 0.25 h, 0.5 h, 1 h, 2 h, 4 h, 6 h, 8 h, 10 h, 12 h, 24 h, and 48 h. Animals in Groups 2–5 were exposed to the test aerosol via inhalation for 120 min at a dose of 18 μg/kg. Serum samples were collected prior to administration and at the following time points after the start of administration: 1 h, 2 h, 2.25 h, 2.5 h, 3 h, 4 h, 6 h, 8 h, 10 h, 12 h, 24 h, and 48 h. Approximately 1 mL of whole blood was collected from the hindlimb vein at each time point and immediately centrifuged at 1500 g for 10 min at room temperature. Blood samples were collected by trained personnel using peripheral venipuncture techniques. Appropriate restraint or sedation was applied as needed to minimize animal stress. The total blood volume collected was controlled within acceptable limits based on body weight and in accordance with institutional animal care guidelines. The resulting serum samples were stored at −65 °C for subsequent analysis. Human IFN-α1b concentrations were quantified using a commercial ELISA kit (Coibo Bio). The assay was applied to serum, Bronchoalveolar lavage fluid (BALF), nasal cavity lavage fluid (NCLF), and tissue homogenates in a fit-for-purpose manner. Matrix effects were evaluated using matrix-matched dilution and calibration approaches, and no significant interference was observed within the quantitative range. Spike-recovery experiments demonstrated acceptable recovery across matrices. Matrix-appropriate lower and upper limits of quantification (LLOQ and ULOQ) were established to ensure reliable measurement of IFN-α1b concentrations. The dose used in cynomolgus monkeys (18 μg/kg) was selected based on prior pharmacokinetic and tissue distribution studies to achieve quantifiable drug exposure in respiratory tissues while maintaining minimal systemic exposure. This exposure-guided dose selection was intended to support evaluation of lung-targeted delivery and safety of inhaled IFN-α1b. All pharmacokinetic and tissue distribution studies in cynomolgus monkeys were conducted at JOINN Laboratories (Suzhou) under GLP conditions and approved by the Institutional Animal Welfare Committee (Approval No.: ACU21-2181).

### Aerosol generation and characterization

Aerosolized IFN-α1b was administered to cynomolgus monkeys using a helmet-based inhalation exposure system under GLP conditions. Aerosol particle size distribution was characterized using a Next Generation Impactor (NGI) at a flow rate of 15 L/min, and mass median aerodynamic diameter (MMAD), geometric standard deviation (GSD), and fine particle fraction (FPF, MMAD < 4 μm) were determined. Across dose groups, aerosols exhibited MMAD values of approximately 1.6–1.8 μm with FPF exceeding 90%. Aerosol concentration was monitored throughout exposure, and the delivered dose was calculated according to the Association of Inhalation Toxicologists (AIT) recommendation using measured aerosol concentration, exposure duration, respiratory minute volume, and body weight. The mean delivered doses were 3.324 ± 0.991, 8.908 ± 2.634, and 23.707 ± 8.422 μg/kg/day for the low-, mid-, and high-dose groups, respectively, corresponding to dose levels selected for evaluation of efficacy, pharmacokinetics, and safety. These parameters were used to support translation of the nominal inhalation dose (18 μg/kg) to the estimated delivered dose in cynomolgus monkeys. This system was used exclusively for aerosol exposure in cynomolgus monkeys.

### Tissue distribution of human IFN-α1b in cynomolgus monkeys

Cynomolgus monkeys in groups 2–4 were anesthetized via intramuscular injection of Zoletil 50 (12 mg/kg) at 3, 6, 26, and 50 h post-drug administration. Euthanasia was performed through femoral artery exsanguination. BALF, NCLF, as well as tissues from the nasal cavity, larynx, trachea, bronchus, left lung, and right lung were collected. Tissues were individually homogenized in five volumes of RIPA premix per gram of tissue. The homogenates were centrifuged at 12,000 RPM for 10 min at 4 °C, and the supernatants were collected and stored at −65 °C for subsequent analysis. Human IFN-*α*1b concentrations were quantified using ELISA (Coibo Bio).

### Repeat dose toxicity study in juvenile cynomolgus monkeys

A total of 50 cynomolgus monkeys (25 females and 25 males, aged 12.2–13.8 months) were randomly assigned to five groups (5 monkeys per gender per group). Group 1 served as the negative control, Group 2 as the solvent control, and Groups 3–5 received low (2 μg/kg/day), medium (6 μg/kg/day), and high (18 μg/kg/day) doses of human IFN-α1b via repeated daily inhalation for 4 weeks. The first day of dosing was designated as Day 1 (D1). Serum samples were collected prior to administration (pre-dosing) and on D1, D15, D29, and D56 for antibody detection against IFN-α1b. Throughout the experiment, body temperature, blood counts, biochemical parameters (UREA, CRE, AST, ALT, CK, LDH), T lymphocyte subsets (CD3+, CD3 + CD4+, CD3 + CD8+), and serum cytokines (IL-2, IL-4, IL-5, IL-6, TNF-α, IFN-*γ*) were monitored. On D29, three monkeys from each group were euthanized, with the remaining two euthanized on D56. Following euthanasia, brain, lung, liver, heart, spleen, and kidney tissues from the high-dose group (18 μg/kg/day) were collected and stained for histopathological analysis. Additionally, a portion of lung and trachea tissue from Groups 2–5 was analyzed for IFN-α1b concentrations. The repeat-dose toxicity study in cynomolgus monkeys was conducted at JOINN Laboratories (Suzhou) under GLP conditions and approved by the Institutional Animal Welfare Committee (Approval No.: ACU21-2177). All procedures adhered to the guidelines and policies set forth by the Animal Care and Use Committee of the respective research units. The overall study design and sampling schedule for cynomolgus monkeys are summarized in Supplementary Table S2.

### Statistical analysis

Data are presented as mean ± SD unless otherwise indicated. Prior to parametric analysis, all data sets were evaluated for normality using the Shapiro–Wilk test and for homogeneity of variances using the Brown-Forsythe test. For *in vitro* antiviral assays, EC₅₀ values and corresponding 95% confidence intervals (CI) were calculated using nonlinear regression with a four-parameter logistic model. *In vivo* lung viral titers were log₁₀ transformed to stabilize variances and satisfy normality requirements before being analyzed via one-way ANOVA followed by Dunnett’s multiple-comparison test. In instances where homogeneity of variances could not be confirmed, the Brown-Forsythe version of ANOVA was employed to ensure the robustness of the statistical conclusions. Effect sizes for viral reduction were expressed as mean differences in log₁₀ PFU/g lung tissue with 95% CI. Pharmacokinetic parameters, including absolute bioavailability and tissue-to-serum exposure ratios, were quantitatively determined using non-compartmental analysis. Toxicity endpoints were evaluated using one-way ANOVA or the Kruskal-Wallis test, as appropriate, with *post hoc* corrections for multiple comparisons. All statistical analyses were performed using GraphPad Prism software, and a *p* value of less than 0.05 was considered statistically significant.

## Results

### The human IFN-α1b showed antiviral activity against RSV *in vitro* and *in vivo*

**Figure 1:**
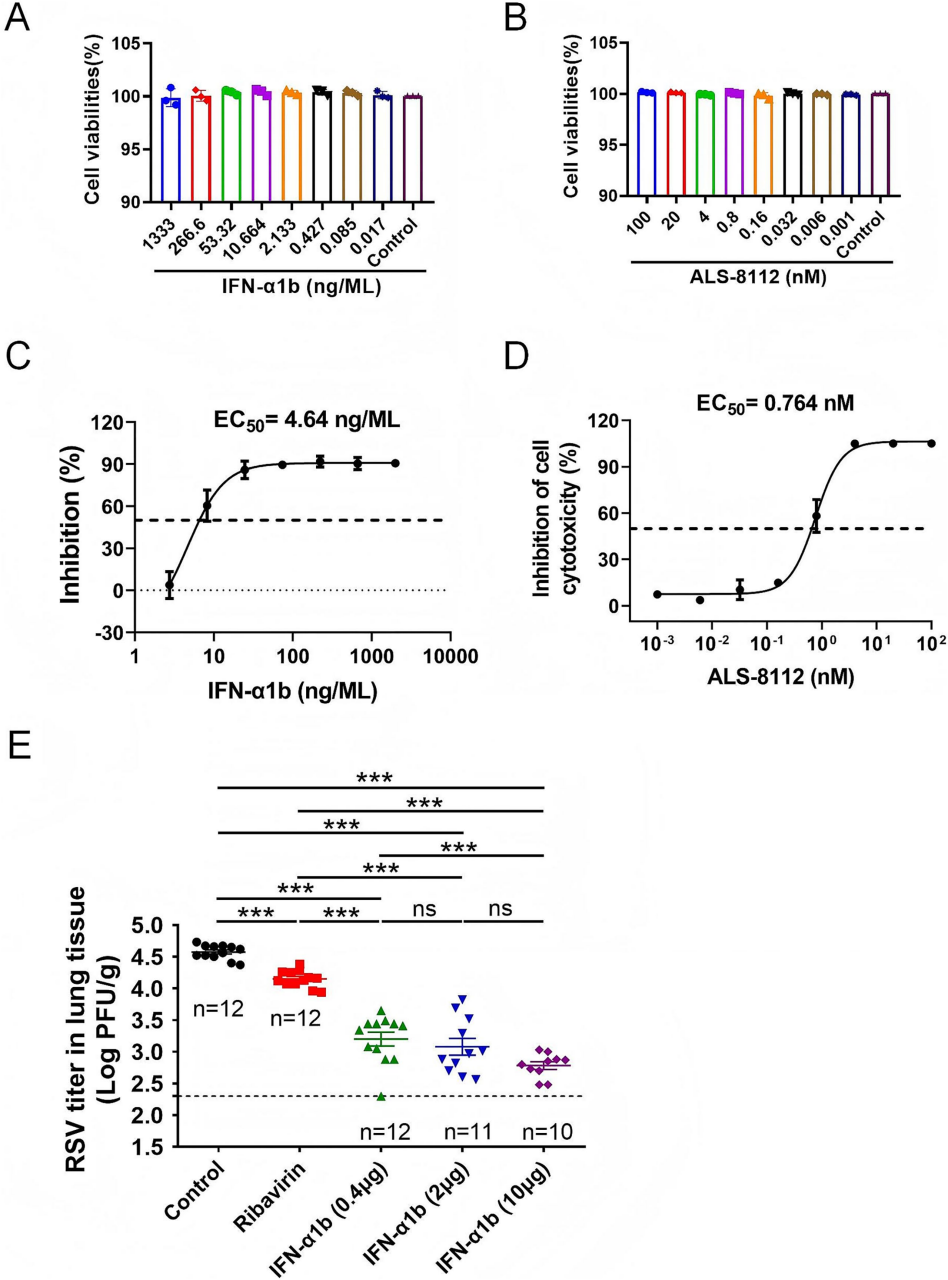
IFN-α1b exhibits antiviral activity against RSV both *in vitro* and *in vivo*. **(A,B)** Cytotoxicity of IFN-α1b **(A)** and ALS-8112 **(B)** in Hep-2 cells measured by CCK-8 assay after 5 days of treatment Data are presented as mean ± SD (*n =* 3 independent experiments). **(C,D)** Antiviral activity of IFN-α1b **(C)** and ALS-8112 **(D)** against RSV A strain Long in Hep-2 cells. EC₅₀ values were calculated using four-parameter nonlinear regression. Data are presented as mean ± SD (*n =* 3). **(E)** Viral titers in lung tissues of RSV-infected mice at 5 dpi were determined by plaque assay and expressed as log₁₀ PFU/g lung tissue. Data are presented as mean ± SD (*n =* 12 for vehicle, ribavirin, and IFN-α1b 0.4 μg groups; *n =* 11 for IFN-α1b 2.0 μg group; *n =* 10 for IFN-α1b 10 μg group). The reduction in n for high-dose groups was due to technical loss during sample processing and plaque assay, not animal mortality. Statistical analysis was performed using one-way ANOVA followed by Dunnett’s multiple comparisons test. **p < 0.01, ***p < 0.001 versus vehicle control.

The antiviral efficacy of human IFN-α1b was assessed using the RSV A strain (Long), with the polymerase inhibitor ALS-8112 for RSV serving as a positive control. Initially, the cytotoxicity of the compounds was evaluated through CCK-8 assays. IFN-α1b displayed minimal cytotoxicity, with 50% cytotoxic concentration (CC_50_) values exceeding 1,333 ng/mL (Figure 1A), while the CC50 of ALS-8112 was greater than 100 μM (Figure 1B). IFN-α1b also exhibited clear antiviral activity against RSVA, achieving a EC_50_ value of approximately 4.64 ng/mL (Figure 1C). As a positive control, ALS-8112 had a EC_50_ value of 0.764 μM (Figure 1D). We next evaluated the in vivo antiviral activity of the test articles administered via intratracheal aerosolization using a microsprayer in a mouse model of RSV infection. Plaque assays were used to quantify RSV titers in lung tissue samples. The results showed that the mean viral titer in the lungs of vehicle-treated mice was 4.573 (log₁₀ PFU/g). In the ribavirin-treated group (1 mg/dose), the mean viral titer significantly decreased to 4.149 (*p* < 0.01). For the IFN-α1b treatment groups, lung viral titers were reduced to 3.20 log₁₀ PFU/g (95% CI: 2.96–3.44, *n =* 12), 3.08 log₁₀ PFU/g (95% CI: 2.78–3.37, *n =* 11), and 2.78 log₁₀ PFU/g (95% CI: 2.65–2.92, *n =* 10) for doses of 0.4, 2, and 10 μg/animal, respectively. Notably, the viral titer in the 10 μg dose group was significantly lower than that in the 0.4 μg dose group, whereas no significant differences were observed between the 2 μg and 0.4 μg dose groups. Importantly, all IFN-α1b dose groups showed significantly greater reductions in lung viral titers compared with ribavirin under the tested conditions (*p* < 0.001) (Figure 1E).

### Aerosol inhalation of human IFN-α1b has favorable pharmacokinetic profiles in the respiratory system of cynomolgus monkeys

**Figure 2:**
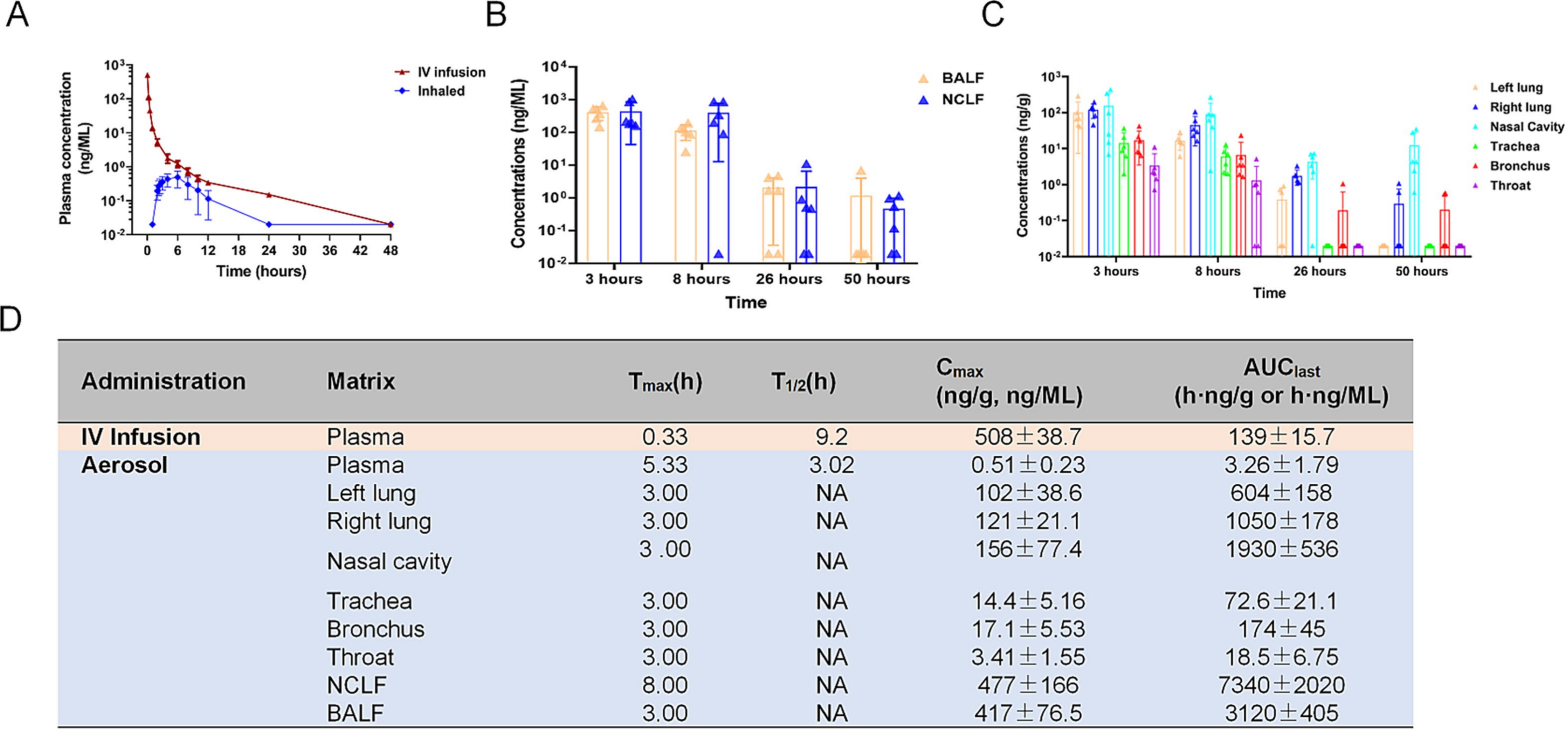
Pharmacokinetic profile of IFN-α1b in cynomolgus monkeys following a single 18 μg/kg dose. **(A)** Serum concentration-time profiles for intravenous and aerosol administration. **(B)** IFN-α1b levels in respiratory lavage fluids at indicated time points. **(C)** Regional tissue distribution in the respiratory tract at 8 and 96 h post-inhalation. **(D)** Comparative pharmacokinetic parameters and exposure ratios (Table), the first row corresponds to IV infusion, and subsequent rows represent aerosol inhalation groups. Data are presented as mean ± SD (*n =* 5 per group). Absolute systemic bioavailability (F%) was calculated to be 3.33% relative to IV administration. Values are presented as mean ± SD. NA: Not calculated due to insufficient data points in the elimination phase for reliable terminal half-life estimation.

Subsequently, we conducted a quantitative evaluation of the pharmacokinetic profiles of IFN-α1b in cynomolgus monkeys after a single intravenous (IV) injection and aerosol inhalation at a dose of 18 μg/kg. In the IV group, the serum concentration of IFN-α1b reached its maximum immediately after administration, with a mean peak concentration (*Cmax*) of 507.83 ng/mL, and thereafter declined over time. In the inhalation group, the serum concentration reached its peak at 6 h post-inhalation (mean: 0.49 ng/mL) and then decreased to a level below the lower limit of quantification (LLOQ) by 24 h (Figure 2A). By comparing the dose-normalized area under the curve (*AUC*0 − 12 h) of the inhalation group to the intravenous group, the absolute systemic bioavailability was calculated to be 3.33%. Furthermore, the concentrations of IFN-α1b in BALF, NCLF, and various tissues, including the left lung, right lung, nasal cavity, trachea, bronchus, and throat, reached their maximum values at 3 h after administration and gradually decreased over time (Figures 2B,C). Notably, the drug exposure in the tissues of the respiratory system was significantly higher than in serum. The order of drug exposure from highest to lowest among tissues and serum was BALF, NCLF, nasal cavity, right lung, left lung, bronchus, trachea, throat, and serum (Figure 2D).

### Continuous and repeated inhalation of human IFN-α1b is safe in juvenile cynomolgus monkeys

**Figure 3:**
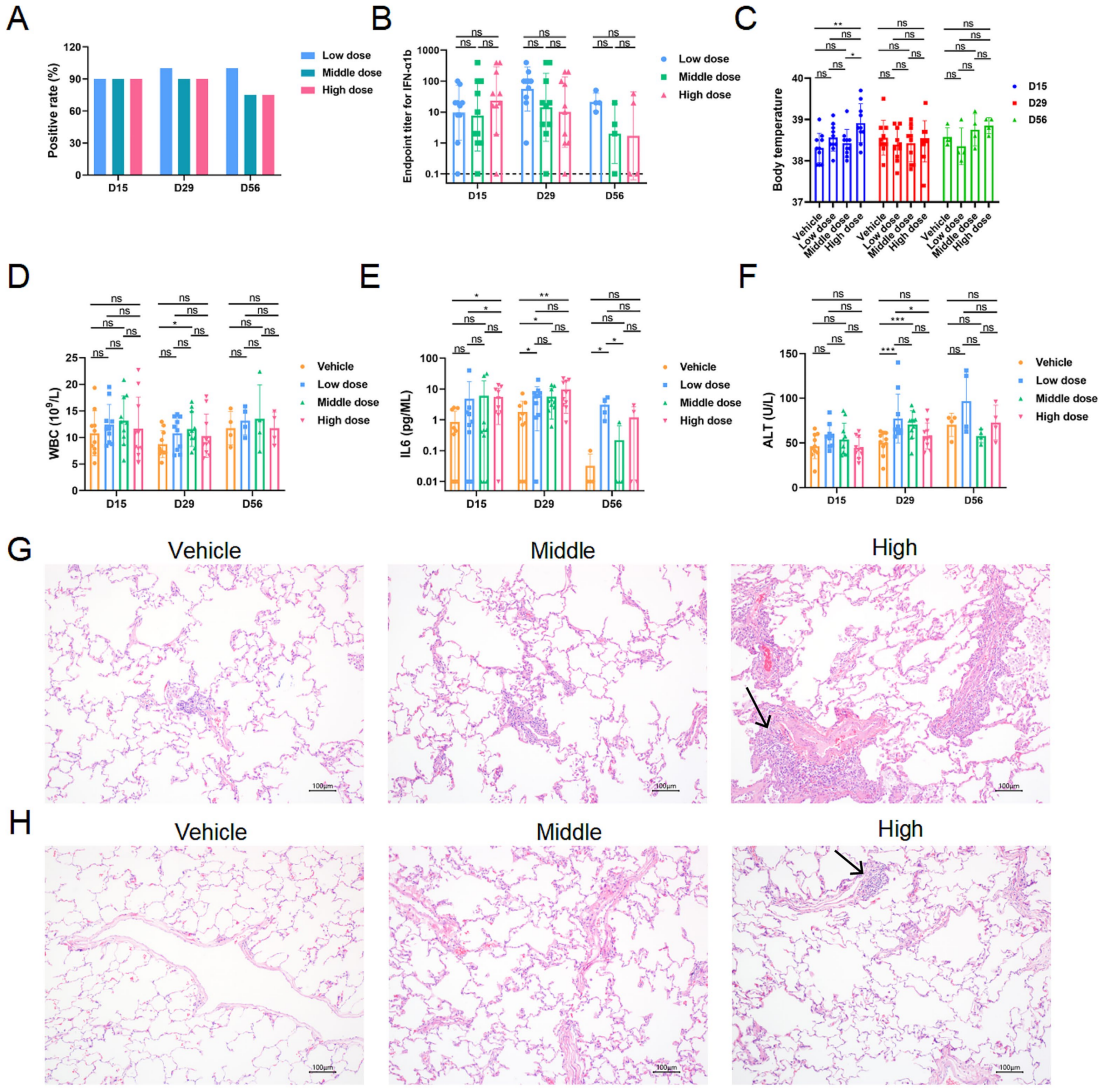
The characteristics of juvenile cynomolgus monkeys after continuous and repeated inhalation of human IFN-α1b for 4 weeks. **(A,B)** Positive rates **(A)** and endpoint titers **(B)** of anti-IFN-α1b antibodies measured at indicated time points during and after repeated inhalation. **(C)** Body temperature monitored throughout the study period. **(D–F)** White blood cell count **(D)**, serum IL-6 concentration **(E)**, and ALT levels **(F)** during and after repeated inhalation. **(G)** Representative histopathological images of lung tissues at Day 29 (hematoxylin and eosin staining, 100×). **(H)** Representative histopathological images of lung tissues at Day 56 (hematoxylin and eosin staining, 100×). Data are presented as mean ± SD (*n =* 10 animals per group). Statistical analysis was performed using one-way ANOVA followed by Dunnett’s multiple comparisons test.

Finally, to evaluate the safety profile of IFN-α1b, juvenile cynomolgus monkeys were exposed to repeated daily inhalations at different doses over a 4-week period. After repeated inhalation of recombinant human IFN-α1b at low (2 μg/kg/day), medium (6 μg/kg/day), and high (18 μg/kg/day) doses, nearly all animals developed anti-drug antibodies (Figure 3A). No significant differences in antibody titer levels were identified across the three dose groups at D15, D29, and D56 (Figure 3B). Throughout the experimental period, no abnormal changes in body temperature were recorded in any group (Figure 3C). After 4 weeks of continuous and repeated inhalation, no substantial abnormalities were detected in blood counts, blood biochemistry, T lymphocyte subsets, or serum cytokines (Figures 3D–F; Supplementary Figure S1), and no mortalities occurred. The central nervous system, cardiovascular system, liver, spleen, and kidneys showed no significant adverse effects, even at the high dose of 18 μg/kg/day (Figure 3; Supplementary Figures S1, S2), although slight and transient elevations in IL-6 (Figure 3E) and CK (Supplementary Figure S1) were observed at D29.

Quantitative histopathological evaluation of the respiratory tissues was performed using Hematoxylin and Eosin (H&E) staining. At D29, macrophage aggregation in the lung alveoli was observed in both the vehicle and medium-dose groups, which was categorized as minimal (Grade 1) severity. In the high-dose group (18 μg/kg/day), findings included both macrophage aggregation and focal perivascular inflammatory cell infiltration (Figure 3G). These inflammatory changes were quantitatively assessed as minimal to mild, ranging from Grade 1 to Grade 2 on a standardized 5-point severity scale, and were not associated with clinical respiratory distress. These findings are consistent with localized innate immune activation rather than toxic tissue injury. Four weeks after the cessation of administration, all monkeys showed significant recovery. Only minimal perivascular inflammatory cell infiltration (Grade 1) remained in a small number of animals from the high-dose group (Figure 3H), confirming the reversible nature of the localized pulmonary response. Detailed quantitative scoring for all observed lesions is provided in Supplementary Table S1.

## Discussion

RSV has remained one of the most significant pathogens causing respiratory diseases globally since its discovery more than 60 years ago (Gatt et al., 2023). Infants, the elderly, and immunocompromised individuals are particularly vulnerable to RSV infection, resulting in substantial morbidity and mortality (Griffiths et al., 2017; Dai et al., 2023). Despite recent advances in RSV vaccines and monoclonal antibodies for prevention, effective antiviral therapies for established RSV infection remain limited (Shi et al., 2017; Kieffer et al., 2022). Thus, there is an ongoing unmet need for safe and effective antiviral strategies targeting RSV.

Previous clinical and experimental studies have demonstrated that higher RSV viral loads are associated with increased disease severity and poorer clinical outcomes (Garcia-Mauriño et al., 2019). These findings highlight viral burden as a key determinant of RSV pathogenesis and a rational therapeutic target. Although monoclonal antibodies such as nirsevimab have shown strong preventive efficacy and are expected to substantially reduce RSV-associated hospitalizations (Sevendal et al., 2024; Garcia-Mauriño et al., 2019), their role is limited to prophylaxis. In contrast, therapeutic options for active RSV infection remain scarce. Ribavirin, the only approved antiviral treatment for RSV, is infrequently used in clinical practice due to concerns regarding toxicity, complex administration, and inconsistent clinical benefit (Shook and Lin, 2017). These limitations underscore the need for alternative therapeutic approaches capable of effectively reducing viral replication during acute RSV infection.

IFNs play a central role in host antiviral defense, and multiple studies have reported impaired IFN responses during RSV infection, particularly in infants. Elevated RSV-induced IgE levels accompanied by reduced IFN-*α*, IFN-*γ*, and IL-2 expression have been observed in children with RSV pneumonia (Gut et al., 2013; Cormier et al., 2014; Sedeyn et al., 2019). Experimental studies further demonstrated that IFN-α administration or adoptive transfer of plasmacytoid dendritic cells prior to RSV infection can attenuate Th2-biased immunopathology and enhance viral clearance in neonatal mouse models (Cormier et al., 2014). However, most prior interferon-based RSV studies relied on systemic or intramuscular administration, which is often associated with limited pulmonary drug exposure and an increased risk of systemic adverse effects. These factors have constrained the broader clinical translation of interferon therapies for RSV. These limitations highlight the importance of alternative delivery strategies to fully realize the therapeutic potential of interferon-based approaches for RSV.

In the present study, aerosolized IFN-α1b demonstrated antiviral activity against RSV in both *in vitro* and *in vivo* models. While antiviral efficacy was evaluated in the murine RSV model, the cynomolgus monkey studies were designed to characterize pharmacokinetic properties, tissue distribution, and safety following inhalation. The repeated dosing regimen in non-human primates enabled the assessment of tolerability and potential local respiratory effects under sustained exposure conditions. The significant reduction in lung viral titers observed in RSV-infected mice, particularly at relatively low inhaled doses, is consistent with known antiviral mechanisms of type I interferons, although these were not directly assessed in this study. Importantly, these findings suggest that localized delivery to the respiratory tract may enhance antiviral efficacy compared with systemic interferon administration reported in earlier studies, highlighting the importance of delivery route in optimizing therapeutic outcomes for respiratory viral infections. It should be noted that the mouse efficacy study was designed to evaluate early intervention during RSV infection, focusing on antiviral activity in the lung rather than therapeutic efficacy at later stages of established disease. In addition, different administration routes were used across species, with intratracheal aerosol delivery in mice to ensure efficient pulmonary deposition and inhalation exposure in non-human primates to better reflect clinically relevant delivery conditions.

Pharmacokinetic and safety evaluations in cynomolgus monkeys are consistent with potential advantages of inhaled IFN-α1b, as aerosol administration achieved high drug exposure in respiratory tissues while maintaining an absolute systemic bioavailability of only 3.33%, indicating that the majority of administered drug remains localized within the respiratory tract. The 18 μg/kg dose used in the monkey studies was selected to represent a significant multiple of the projected clinical dose, providing a robust safety margin and ensuring that drug concentrations remained within quantifiable ranges for a rigorous evaluation of the maximum feasible exposure. Although consistent induction of anti-drug antibodies was observed across dose groups, no dose-dependent adverse clinical effects were detected, suggesting acceptable tolerability within the study period in the non-human primate model. While formal functional neutralization assays were not performed, the presence of these antibodies did not correlate with reduced systemic exposure, altered tissue distribution, or clinically meaningful abnormalities. This suggests that the antibodies did not significantly alter the pharmacokinetic profile or clinical safety markers during the study period, although the potential for these antibodies to diminish biological activity cannot be entirely excluded.

Regarding the localized safety profile, the histopathological changes observed at the 18 μg/kg dose were focal and quantitatively assessed as minimal to mild, ranging from Grade 1 to 2 on a standardized 5-point scale. These findings were fully reversible and were not associated with clinical respiratory impairment or functional deficits. While a definitive safety comparison would require a head-to-head study under identical experimental conditions, our findings highlight the localized nature of inhaled IFN-α1b, which may help reduce systemic exposure compared with systemically administered therapies. Furthermore, although the interaction between drug-induced low-grade inflammation and the complex immunopathology of an active RSV infection was not directly examined in the healthy primate model, our murine efficacy data demonstrated significant viral reduction without evidence of exacerbated lung injury. This suggests that the transient inflammatory response observed here is unlikely to compromise the overall therapeutic benefit, though this balance remains a critical consideration for future clinical monitoring.

Unlike antiviral agents targeting RSV structural or nonstructural proteins, such as F protein or RNA-dependent RNA polymerase inhibitors (Shook and Lin, 2017), type I interferons exert broad antiviral activity that is less susceptible to viral variation. In the present study, antiviral activity was evaluated using the RSV A strain Long as a representative laboratory strain, and broader activity across RSV subtypes and clinical isolates remains to be confirmed in future studies. Inhalation delivery may additionally facilitate rapid activation of mucosal innate immune responses, which are critical for early control of RSV replication in the respiratory tract. These characteristics suggest that aerosolized IFN-α1b may represent a mechanistically distinct therapeutic approach for RSV infection.

It is also essential to consider aerosolized IFN-α1b within the evolving RSV therapeutic landscape, which includes several direct-acting antivirals (DAAs) currently in development. For instance, oral nucleoside analogs such as ALS-8176 and inhaled polymerase inhibitors such as PC786 have demonstrated significant antiviral activity by specifically targeting the viral replication machinery (Wang et al., 2015). However, DAAs focusing on specific viral targets including the fusion (F) protein or polymerase are often susceptible to the emergence of drug-resistant mutations, which can limit their clinical utility across diverse isolates (Cockerill et al., 2019; Coates et al., 2017). In contrast, IFN-α1b exerts its effects by activating a broad repertoire of host-encoded interferon-stimulated genes that interfere with multiple stages of the viral life cycle (Samuel, 2001). This host-centered mechanism potentially offers a greater breadth of activity against various clinical genotypes and a higher barrier to viral resistance compared to protein-specific inhibitors. Furthermore, our aerosolized delivery approach directly addresses the localized nature of RSV infection, representing a potential therapeutic strategy that could complement current standard supportive care or future DAA combinations.

Although our data clearly demonstrate the antiviral efficacy of aerosolized IFN-α1b, the precise underlying mechanisms remain to be fully elucidated. These potential pathways include the direct induction of an antiviral state in epithelial cells as well as broader immunomodulation. Based on the rapid reduction in viral titers observed at peak replication, we speculate that aerosolized IFN-α1b may act, at least in part, through localized induction of interferon-stimulated genes (ISGs) within airway epithelial cells, thereby restricting early viral amplification rather than through systemic immune modulation. Future studies incorporating transcriptomic profiling of respiratory tissues will be required to delineate these mechanisms in detail. Furthermore, given that RSV-induced immunopathology is often associated with a skewed Th2 response, the potential for inhaled IFN-α1b to recalibrate the host response toward a protective Th1 phenotype represents a compelling hypothesis that warrants further investigation.

This study has several limitations. First, antiviral efficacy was evaluated in a murine model using a pre-infection dosing regimen and a single time point (5 dpi), which does not fully reflect clinically relevant treatment settings and does not allow assessment of early viral kinetics or confirmation of comparable initial infection levels across groups. In addition, species differences, including the limited cross-reactivity of human IFN-α1b in mice (Guerrero-Plata et al., 2005; Goritzka et al., 2014; Hijano et al., 2018), the absence of lung pharmacokinetic data, and the proof-of-concept nature of the model limit direct translational interpretation. The observed dose–response relationship may therefore not directly predict clinical outcomes. Second, although cynomolgus monkeys provide a relevant model for pharmacokinetics and safety, the limited sample size, lack of correction for lavage dilution, and absence of functional characterization of anti-drug antibodies constrain interpretation. Differences in immune maturity and the long-term impact of immunogenicity also remain to be determined. Third, mechanistic insights were not directly investigated, as key molecular readouts such as ISG induction and downstream signaling pathways were not assessed, limiting detailed interpretation of the antiviral mechanism. These limitations highlight the need for future studies incorporating delayed-treatment models, viral kinetic analysis, and molecular characterization to further define the therapeutic potential of aerosolized IFN-α1b.

## Conclusion

This study adopted a mouse model infected with RSV to evaluate the therapeutic effect of human IFN-α1b *in vivo*. Through single-dose inhalation and intravenous injection administrations in cynomolgus monkeys, the pharmacokinetic characteristics of human IFN-α1b were systematically evaluated. Meanwhile, after inhalation administration, the distribution of the drug in lung tissues was detected, and its inhalation toxicity was evaluated through repeated administrations. These findings suggest that inhaled IFN-α1b has three potentially advantageous attributes for RSV treatment: (1) low nanomolar potency *in vitro*, (2) dose-dependent viral clearance *in vivo*, and (3) localized delivery with minimal systemic toxicity. Given its low production cost and compatibility with existing nebulizers, this approach warrants further clinical evaluation, particularly in settings where cost-effective interventions are needed and RSV burden is highest.

## Data Availability

The original contributions presented in the study are included in the article/Supplementary material, further inquiries can be directed to the corresponding authors.

## Supplementary material

SUPPLEMENTARY FIGURE S1 Blood biochemistry and lymphocyte profiles in juvenile cynomolgus monkeys after 4-week repeated inhalation of human IFN-α1b. **(A,B)** CD4⁺ **(A)** and CD8⁺ **(B)** T lymphocyte subsets expressed as percentages of total lymphocytes. (C–G) Blood biochemical parameters including urea **(C)**, creatinine **(D)**, AST **(E)**, CK **(F)**, and LDH **(G)** measured at indicated time points. Data are presented as mean ± SD (n = 10 animals per group). Statistical analysis was performed using one-way ANOVA followed by Dunnett’s multiple comparisons test.

SUPPLEMENTARY FIGURE S2 Representative histopathological sections of respiratory tissues. Representative Hematoxylin and Eosin (H&E) images of brain **(A,B)**, heart **(C,D)**, liver **(E,F)**, spleen **(G,H)**, and kidney **(I,J)** tissues from vehicle-treated and high-dose IFN-α1b-treated cynomolgus monkeys. Images were acquired at 100× magnification.
