# Supplementary figures and images for: Aerosol inhalation of human IFN-α1b exhibits anti-RSV activity in mice and favorable pharmacokinetics/safety in cynomolgus monkeys
Source: Front Microbiol. 2026 Apr 30;17:1767720. doi: 10.3389/fmicb.2026.1767720 (PMC13171780; doi:10.3389/fmicb.2026.1767720)

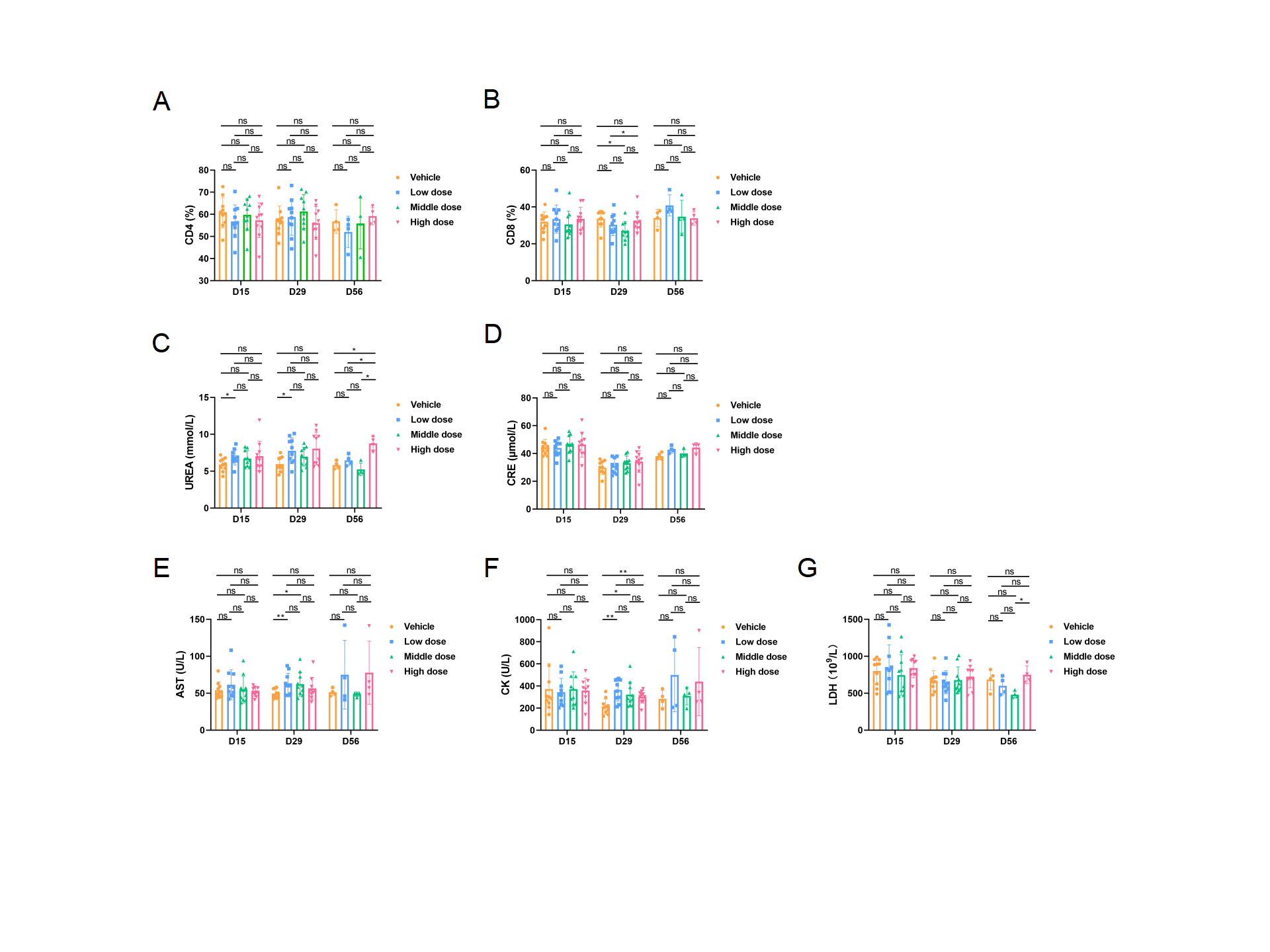

Supplement: SUPPLEMENTARY FIGURE S1 — Blood biochemistry and lymphocyte profiles in juvenile cynomolgus monkeys after 4-week repeated inhalation of human IFN-α1b. (A,B) CD4⁺ (A) and CD8⁺ (B) T lymphocyte subsets expressed as percentages of total lymphocytes. (C–G) Blood biochemical parameters including urea (C), creatinine (D), AST (E), CK (F), and LDH (G) measured at indicated time points. Data are presented as mean ± SD (n = 10 animals per group). Statistical analysis was performed using one-way ANOVA followed by Dunnett’s multiple comparisons test. [file Image_1.tif]

A

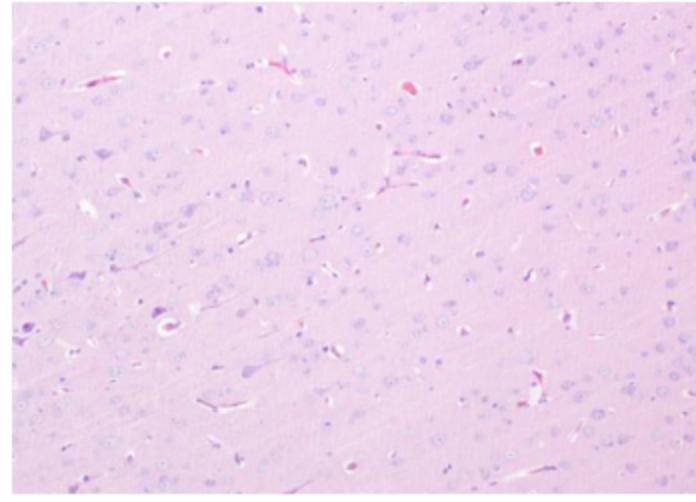

B

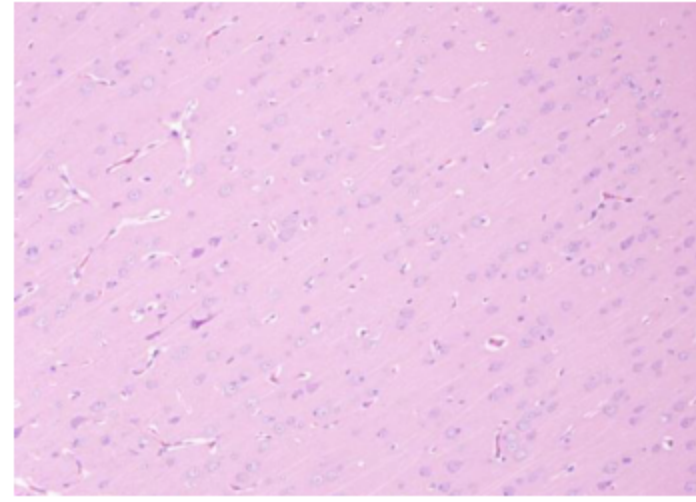

C

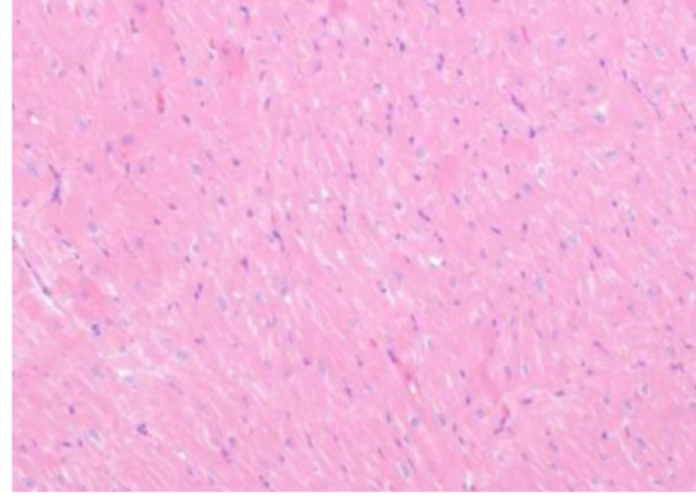

D

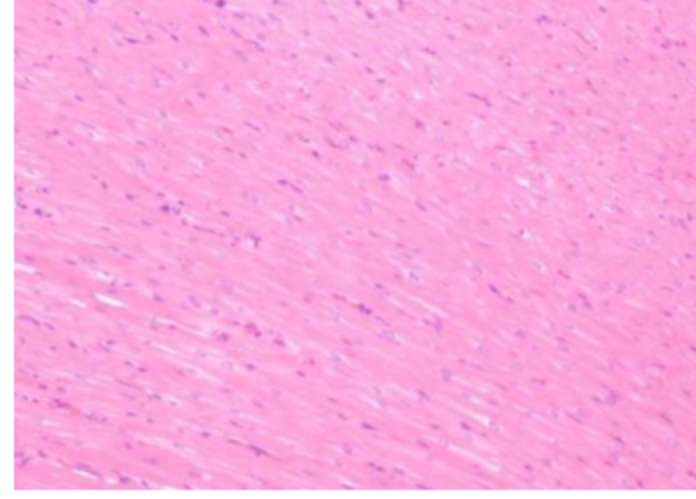

E

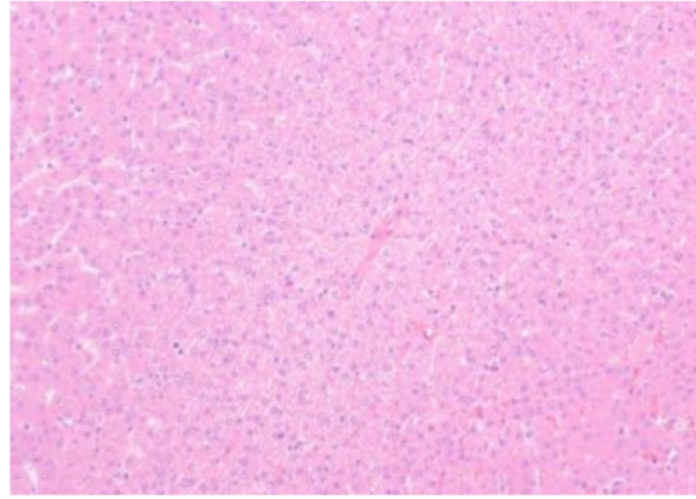

F

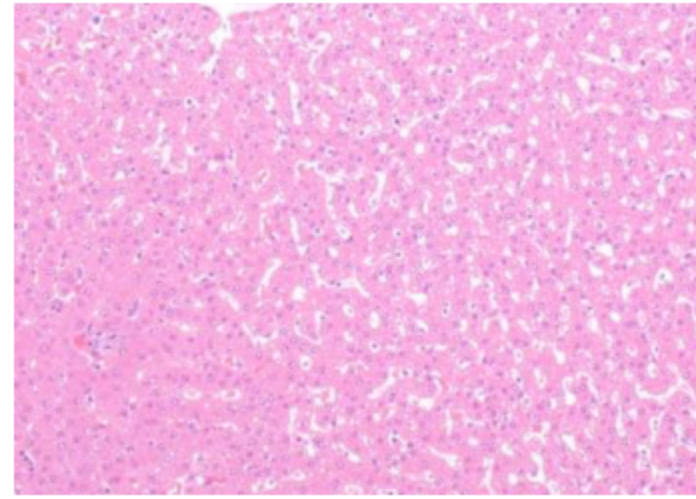

G

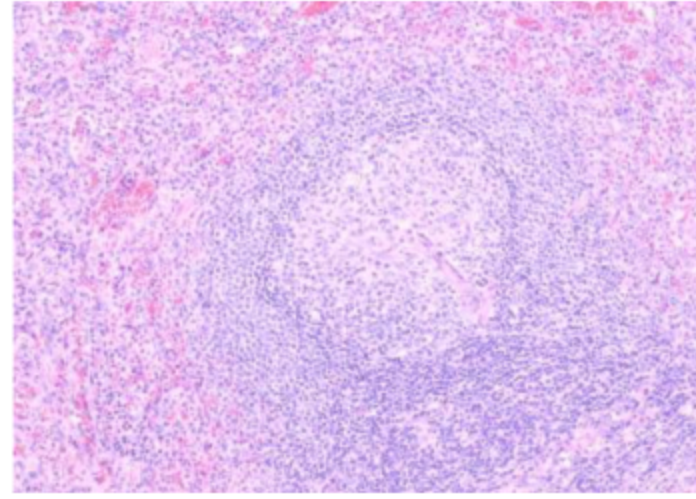

H

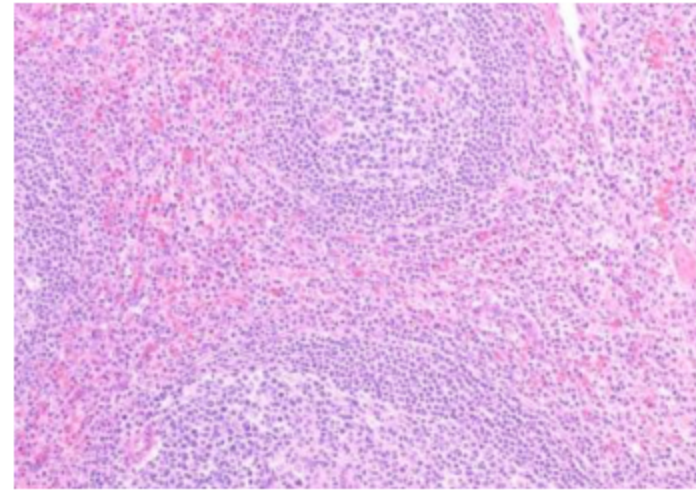

I

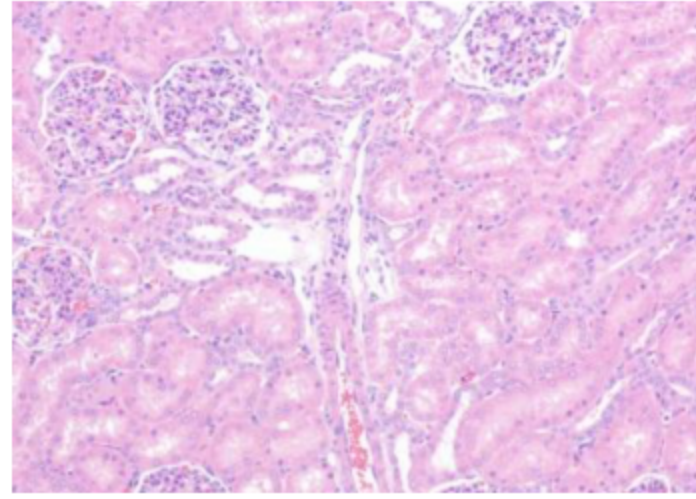

J

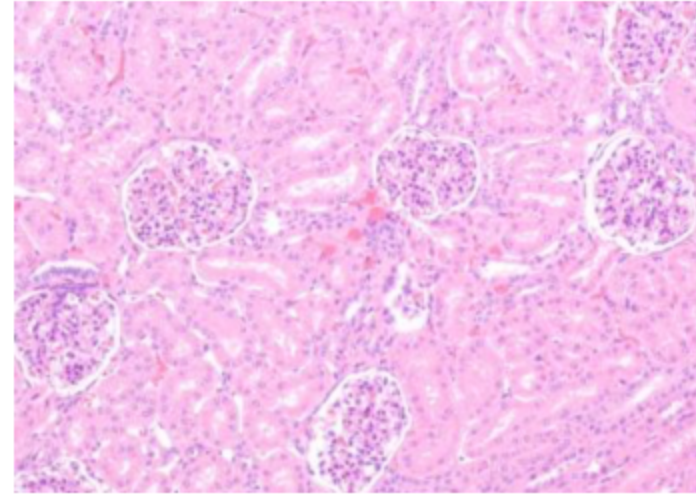

Supplement: SUPPLEMENTARY FIGURE S2 — Representative histopathological sections of respiratory tissues. Representative Hematoxylin and Eosin (H&E) images of brain (A,B), heart (C,D), liver (E,F), spleen (G,H), and kidney (I,J) tissues from vehicle-treated and high-dose IFN-α1b-treated cynomolgus monkeys. Images were acquired at 100× magnification. [file Data_Sheet_1.pdf]
