# Supplementary material for: Aerosol inhalation of human IFN-α1b exhibits anti-RSV activity in mice and favorable pharmacokinetics/safety in cynomolgus monkeys
Source: Front Microbiol. 2026 Apr 30;17:1767720. doi: 10.3389/fmicb.2026.1767720 (PMC13171780; doi:10.3389/fmicb.2026.1767720)
Supplement: Supplementary file 3 [file Table_1.docx]

Table S1. Quantitative Histopathological Scoring of Respiratory Tissues in Cynomolgus Monkeys

| Organ/Tissue | Finding | Severity Grade (0 to 5) | Frequency (18 μg/kg group) |
| --- | --- | --- | --- |
| Lung (All lobes) | Interstitial inflammatory cell infiltration | 1 to 2 (Minimal to Mild) | 2 out of 10 animals |
|  | Alveolar septal thickening | 1 (Minimal) |  |
| Nasal Cavity | Focal mucosal inflammation | 1 (Minimal) | 1 out of 10 animals |
| Trachea | No significant abnormalities | 0 (None) | 0 out of 10 animals |
