# Supplementary material for: Aerosol inhalation of human IFN-α1b exhibits anti-RSV activity in mice and favorable pharmacokinetics/safety in cynomolgus monkeys
Source: Front Microbiol. 2026 Apr 30;17:1767720. doi: 10.3389/fmicb.2026.1767720 (PMC13171780; doi:10.3389/fmicb.2026.1767720)
Supplement: Supplementary file 4 [file Table_2.docx]

# Table S2. Study design and sampling schedule in cynomolgus monkeys

## (A) Pharmacokinetic and tissue distribution study

| Study component | Group | Dose | Sampling type | Time points |
| --- | --- | --- | --- | --- |
| PK (serum) | IV | 18 μg/kg | Blood | Pre, 2 min, 0.25, 0.5, 1, 2, 4, 6, 8, 10, 12, 24, 48 h |
| PK (serum) | Inhalation | 18 μg/kg | Blood | Pre, 1, 2, 2.25, 2.5, 3, 4, 6, 8, 10, 12, 24, 48 h |
| Tissue distribution | Inhalation groups (Groups 2–4) | 18 μg/kg | BALF, NCLF, tissues | 3, 6, 26, 50 h |

## (B) Repeat-dose toxicity study

| Study component | Group | Dose | Sampling type | Time points |
| --- | --- | --- | --- | --- |
| Repeated dosing | Control / Low / Mid / High | 0 / 2 / 6 / 18 μg/kg/day | Inhalation (daily) | Day 1–28 |
| ADA | All groups | - | Serum | Pre, D1, D15, D29, D56 |
| Clinical pathology | All groups | - | Blood | Pre, D1, D15, D29, D56 |
| Necropsy | All groups | - | Tissue collection | D29 (partial), D56 (recovery) |
